# Antibiotics (Macrolides and Lincosamides) Consumption Trends and Patterns in China’s Healthcare Institutes. Based on a 3 Year Procurement Records, 2015–2017

**DOI:** 10.3390/ijerph18010113

**Published:** 2020-12-26

**Authors:** Wenchen Liu, Ali Hassan Gillani, Sen Xu, Chen Chen, Jie Chang, Caijun Yang, Wenjing Ji, Minghuan Jiang, Mingyue Zhao, Yu Fang

**Affiliations:** 1 Department of Pharmacy Administration and Clinical Pharmacy, School of Pharmacy, Xi’an Jiaotong University, Xi’an 710061, China; liuwenchen@stu.xjtu.edu.cn (W.L.); hassangillaniali@yahoo.com (A.H.G.); marsxs@stu.xjtu.edu.cn (S.X.); chenchen00@stu.xjtu.edu.cn (C.C.); yangcj@xjtu.edu.cn (C.Y.); wenjing.ji@xjtu.edu.cn (W.J.); jiangmh2017@xjtu.edu.cn (M.J.); mingyue0204@xjtu.edu.cn (M.Z.); 2 Center for Drug Safety and Policy Research, Xi’an Jiaotong University, Xi’an 710061, China; 3 Shaanxi Center for Health Reform and Development Research, Xi’an Jiaotong University, Xi’an 710061, China; 4 Research Institute for Drug Safety and Monitoring, Institute of Pharmaceutical Science and Technology, China’s Western Technological Innovation Harbor, Xi’an 710049, China

**Keywords:** antibiotic use, macrolides, lincosamides, trends, patterns, China

## Abstract

In this study, we investigated the trends and patterns of antibiotic consumption (macrolides and lincosamides) in China’s healthcare institutions from 2015 to 2017. The China Drug Supply Information Platform (CDSIP) was officially launched in 2015. We collected records from this national centralized bidding procurement system between 2015 and 2017. The use of J01F antibiotics (macrolides or lincosamides) was calculated in a defined daily dose per 1000 inhabitants per day (DID).Purchase data from 70,366 national medical facilities included in the CDSIP were collected. The procurement data of 66,007 medical facilities have not changed over 3 years. There is a slight decline in the consumption of J01F antibiotics, which decreased from 3.03 DID in 2015 to 2.91 DID in 2017. Azithromycin (20.6%) was the most commonly used antibiotic in 2017 among all classes, followed by clindamycin (17.9%) and erythromycin (13.7%). Parenteral antibiotics accounted for 32.0% of total antibiotic consumption and 59.6% of total antibiotics expenditure in 2017. The overall consumption of most antibiotics decreased slightly over the 3-yearstudy period. This may be owing to China’s health-related policies in the past few years. A gap still exists in antibiotic use between regions and dosage forms. Further studies are needed to optimize antibiotic prescribing and reduce antibiotic resistance.

## 1. Introduction

Low- and middle-income countries are highly burdened with the problem of antibiotics overuse, which is strongly associated with antimicrobial resistance (AMR) [1,2]. Deaths due to antimicrobial-resistant infections reach 700,000 annually, and may exceed 10 million by 2050 and impact the global economy, with estimated losses of USD 100 trillion, if the increasing trend is not properly contained [3]. The worldwide use of antibiotics increased by 39% between 2000 and 2015 [4], with most being consumed in primary healthcare institutions [5].

In China between 2000 and 2015, the consumption of antimicrobials increased at a rate of 82.6%, with 2.3 to 4.2 billion cumulative defined daily doses (DDDs) [4]. China is considered to be the second largest consumer of antibiotics worldwide. Considering that the country currently has the world’s largest population, China is endowed with the responsibility to constrain the use and abuse of antibiotics [6]. The average prescription rate of antibiotics is 72% [7], and 7 in 10 Chinese inpatients are given antibiotics [8], which is a noteworthy statistic.

In other parts of the world, surveillance plays an important role in limiting the overuse of antimicrobials [9,10,11]. Over the past few years, the Chinese government has begun taking measures to improve the regulation of antibiotic use [12]. These efforts include a series of measures, such as the establishment of national guidelines in 2004 [13] and the initiation of AMR and antibiotic use surveillance networks in 2005 [14]. A 3-year national-level regulatory campaign was launched in 2011 to control total antibiotic use in secondary and tertiary hospitals [15]. This campaign was legislated as a ministerial decree in 2012 [16].The Chinese government established new administrative rules for the clinical use of antibiotics in 2012, and these rules are believed to be the strictest regulatory checks on prescription antibiotics to date [16,17]. Three categories of antibiotics were set forth: restricted, non-restricted, and controlled. Controlled antibiotics are not included in the Essential Medicines List for primary care hospitals. Prescriptions of restricted or controlled antibiotics are subject to strict administrative restrictions, and penalties are applied for violating the regulations [18].

Despite these strict policies and legislation, there are increases in the trend of antibiotics use in primary [2] and tertiary care institutes of China [6]. Thus far, studies have focused on the general use of antibiotics, and no studies have investigated the specific classes of antibiotics for all of China. Our study thus focused on lincosamides and macrolides consumption in the primary, secondary, and tertiary care hospitals of 28 provinces (autonomous regions and municipalities) in China.

## 2. Method

### 2.1. Study Design and Setting

China occupies 9.6 million square kilometers, with a total population of 1.4 billion [19,20]. In 2017, the gross domestic product (GDP) was RMB 827.13 billion, with an annual per capita GDP of RMB 59,660 [20]. Total national health spending reached RMB 5159.88 billion, and per capita health spending was RMB 3712.2, accounting for 6.2% of GDP in 2017. The Chinese Drug Supply Information Platform (CDSIP) was officially launched in 2015. Health facilities upload information daily on ordering, storing and delivering, and medicines settlement to the CDSIP. The government can monitor prices, quantities, distribution and warehousing, and can organize medications purchased by medical institutions and strengthen management based on relevant information. As of 2017, the CDSIP covered more than 80% of drug purchasing data from national health facilities. The CDSIP is the most appropriate and comprehensive information platform for national drug purchasing data. We only include procurement records for systemic-use antibiotics; topical-use antibiotics records were not included for analysis.

### 2.2. Data Collection

In this study, we collected purchasing data for macrolides or lincosamides from 70,366 national medical facilities included in the CDSIP, and conducted a cross-sectional study of the current state of antibiotic use in China. Procurement data for 66,007 medical facilities that have not changed over 3 years were collected for longitudinal analysis of antibiotic use from 2015 to 2017.

The procurement information extracted from the CDSIP includes the name of the province (autonomous regions and municipalities), the name of the drug, the level of healthcare institution, dosage form, package price, strength, quantity of packs, manufacturer, and ordering time. We collected data for two subclasses of the main classes, including the following antibiotics: erythromycin, spiramycin, midecamycin, roxithromycin, josamycin, clarithromycin, azithromycin, dirithromycin, ambroxol, acetylkitasamycin, kitasamycin, clindamycin, and lincomycin. Healthcare institutions names were not disclosed to protect privacy. Healthcare institutions were sampled hierarchically based on geographical and socioeconomic factors. We selected primary hospitals, secondary hospitals, and tertiary-level hospitals in 28 provinces (autonomous regions and municipalities), on the basis of having full records of antibiotic use during the 3-year study period. Regional variation in antibiotic use was calculated by dividing the country into regions: North China (Beijing, Tianjin, Hebei, Shanxi, Neimeng), Northeast China (Liaoning, Jilin, Heilongjiang), Eastern China (Shanghai, Jiangsu, Zhejiang, Anhui, Fujian, Jiangxi, Shandong),Central China (Henan, Hubei, Hunan), Southern China (Guangdong, Guangxi, Hainan), Southwest China (Chongqing, Sichuan, Guizhou, Yunan, Xizang), and Northwest China (Shaanxi, Gansu, Qinghai, Ningxia, Xinjiang).

### 2.3. Data Management and Analysis

Antibiotic sales records were identified according to the Anatomical Therapeutic and Chemical (ATC) classification J01 (i.e., antibacterial for systemic use), with DDD as the unit of measurement, as recommended by the World Health Organization (WHO) collaborating center for drug statistic methodology [21]. A total of 13 unique chemical substances were identified in combination or single antibiotics. These antibiotics were grouped into two ATC-4 classes.

Sales of antibiotics were calculated using the DDD method developed by the WHO [21]. DDD represents the daily average maintenance dose for a specific drug that is used in adults for its main indication. The antibiotic use data were then converted into DID at the level of the active substance, to detect trends in annual antibiotic sales.

## 3. Results

The total antibiotic consumption of J01F antibiotics generally decreased from 3.03 DID in 2015 to 2.91 DID in 2017. However, the average costs for antibiotics increased from RMB 203,756.50 in 2015 to RMB 218,573.20 in 2017. The regional distribution of antibiotic consumption varied considerably in 2017 from a minimum 1.25 DID in the Northwest region to a maximum 5.47 DID in Central China.

### Patterns of Antibiotic Use

**Figure 1:**
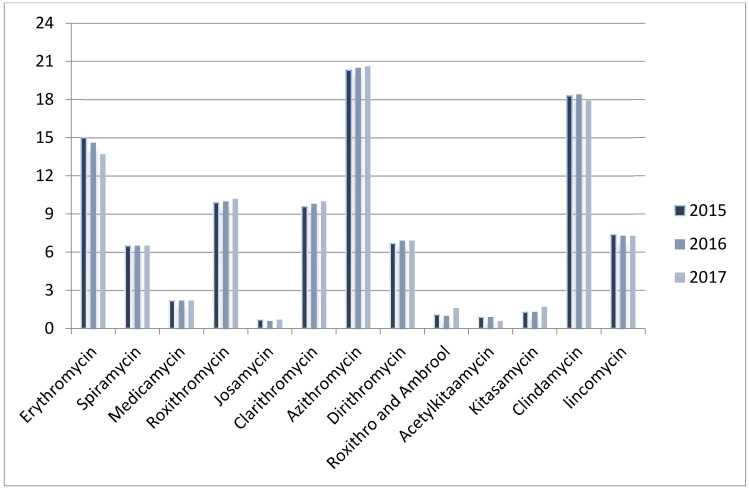
% Changes in the patterns of J01F use in hospital care between 2015 and 2017.

The most commonly used J01F antibiotic was azithromycin, which accounted for 20.6% of total use in the selected healthcare institutions in 2017, 20.5% in 2016 and 20.3% in 2015. This was followed by clindamycin, for which the consumption was 17.9% in 2017, 18.4% in 2016 and 18.3% in 2015.Josamycin was the least frequently used in healthcare institutions, accounting for 0.7% in 2017, 0.6% in 2016 and 0.7% in 2015 of the total antibiotic consumption (Figure 1).

During our study period, the consumption of azithromycin decreased from 5.04 DID to 4.83 DID. However, consumption of clarithromycin and roxithromycin increased from 5.01 DID to 5.15 DID, and from 7.76 DID to 8.03 DID, respectively.

**Figure 2:**
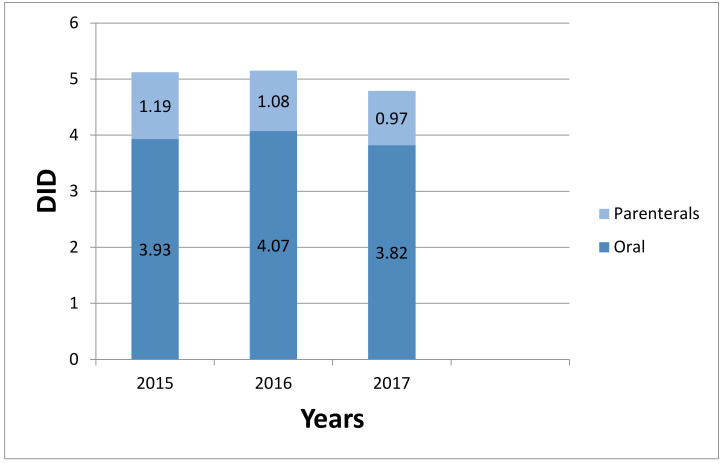
Consumption of oral and parenteral antibiotics in China, 2015–2017.

The total parenteral use of antibiotics in 2015 was 1.19 DID, which accounted for 32.9% of total antibiotics use, and oral antibiotics consumption was 3.93 DID, which is 67.1% of total antibiotics use. However, there was a decrease in parenteral consumptionto 0.97 DID in 2017, accounting for 32% of total antibiotic consumption, and oral antibiotic use decreased to3.82 DID (68%) (Figure 2). The expenditure for parenteral antibiotics accounted for 60.7% of total antibiotics expenditure in the study period, and this varied significantly from 62.0% in 2015 to 59.6% in 2017 (Table 1).

**Figure 3:**
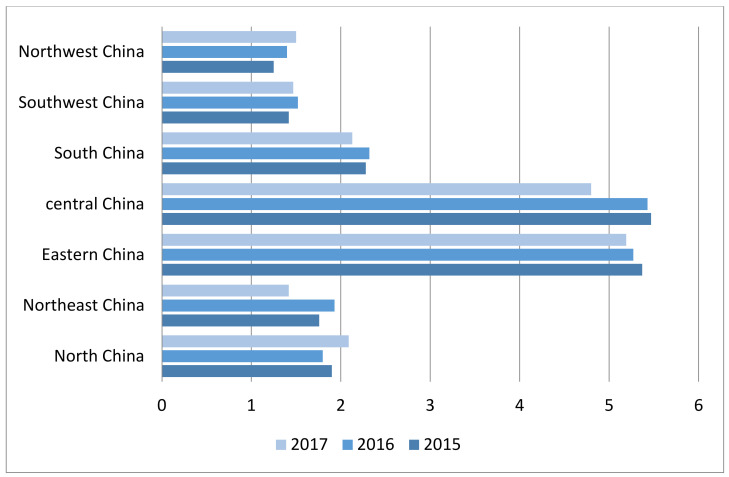
Antibiotic consumption in DID in different regions of China from 2015 and 2017.

Considering the regional variation, the consumption of antibiotics was highest in Central China, and this is followed by the Eastern China region. The consumption of antibiotic decreased from 5.47 DID in 2015 to 4.80 DID in 2017 in the Central Chinaregion, and from 5.378 DID in 2015 to 5.19 DID in 2017 in the Eastern region. However, there was an increase in the antibiotic consumption over time in North China and Northwest China region. Usage increased from 1.90 DID in 2015 to 2.09 DID in the North China region, and from 1.25 DID in 2015 to 1.51 DID in 2017 in the Northwest region (Figure 3).

## 4. Discussion

This is the first national population-based study using the ATC/DDD methodology of the WHO to evaluate the use of J01F antibiotics in China. Our findings revealed the latest patterns and trends in antibiotic use since the creation of the CDSIP in the country. Our study estimated the change in antibiotic consumption over a 3-year period, providing the opportunity to benchmark gaps in antibiotic use in China. In reforming the Chinese health system, the state council proposed the creation of an effective shared medical and health information system, creating an information platform to enhance the oversight of the entire drug-buying process and to ensure drug quality. The creation of the CDSIP broke down information silos and achieved interconnection among information, data and resource exchanges, such as the central provincial drug delivery platform, hospitals, health insurance institutions, and pricing authorities. Population-based drug-use surveys based on these health management data can provide strong and evidence-based support for medication management, and are instrumental in improving drug safety for the general public.

The consumption of antibiotics is a main factor in the development of AMR [22]. However, we identified a decrease in the use of antibiotics over the 3-year study period (Table 1).These results are in accordance with 6-year surveillance data in Shandong Province, showing a decrease in macrolide consumption but a slight increase in the use of lincosamides [23].This decrease reflects the strict implementation of the country’s health-related policies. In early 2011, special rectification with respect to antibacterial drugs was conducted nationwide, laying a solid foundation for the rational use of antibacterial agents. Published in 2015, the “Guidelines for the Clinical Application of Antimicrobials” clarify the basic principles for the administration of antibacterial drugs, highlighting the indications and precautions for various antibacterial agents, as well as the treatment of numerous bacterial infections and principles in the treatment of pathogens. At the same time, the “Requirements for Evaluating the Management of Indicators and Clinical Applications of Antibacterial Drugs”have been revised and improved, stipulating that the number of antimicrobials used in secondary and tertiary hospitals’ catalogs must not exceed 35 and 50, respectively [24]. The function of administrative health management is to examine, inspect, and evaluate medical establishments according to relevant assessment indicators. All this can explain the continued decline in antibiotic use in China.

With respect to regional use, the highest was seen in central China, and this decreased slightly from 2015 to 2017. These results were inconsistent with previous studies from China, where the highest consumption was seen in the eastern region. This can best be explained in terms of patient flow between provinces, which is caused by unbalanced healthcare resource allowance. Additionally, owing to the short geographical distance between the central and eastern regions, this phenomenon may be more common in Central China than in the west [6].

Total parenteral use declined non-significantly from 2015 to 2017. The rate of use of antibiotics via parenteral administration was much lower than in a previous study from China [23]. This high incidence of injectable use in a previous study may be related to the ease of practice among physicians and the high demand of patients [25]. However, a decrease in injectable antibiotics use has been seen in four provinces of China, owing to the strict implementation of the National Essential Medicines Policy (NEMP) [26].Public health interventions and education are set up to promote the proper use of antibiotics [27]. Total expenditure for parenteral antibiotics decreased to 59.6% in 2017 from 62.0% in 2015. With implementation of the NEMP, the price did not decline, even with the price control policy of the National Essential Medicines System [28]. This outcome might be partly caused by patients being treated with too many medicines, especially antibiotics. These results confirm that the medical costs for each prescription that includes antibiotics or injections are higher than those without antibiotics or injections, which is consistent with other studies [29]. Moreover, a report from three provinces in China previously showed that some manufacturers have ceased the production of certain essential drugs because the recommended price controls and procurement procedures in the NEMS substantially reduced their profits. That report also showed that most health care providers complained of price hikes for some drugs that were lower-priced before the establishment of the NEMS [30]. These factors may increase medical expenses. Furthermore, owing to widespread corruption in the procurement process, economic incentives provided to hospitals, manufacturers, and physicians may also lead to the more frequent use of more expensive drugs [31,32,33]. If profits from the sale of drugs are eliminated, appropriate financial compensation mechanisms are needed to ensure the sustainable development of primary care facilities, as income from medicines in the past exceeded 50% of the income in Chinese hospitals [34].

At present, antibiotics use in the medical institutions of China is strictly controlled. However, the practice of non-prescription dispensing of antibioticsin the community still exists, requiring regulatory intervention, professional training, and public health education [35,36] At the same time, the monitoring of antibiotic use should be carried out in conjunction with drug resistance monitoring and its adverse reactions, with the participation of infectious disease physicians, microbiologists, and pharmacists, who play an important role in the rational use of antibiotics [37,38].

This study has some strengths and some limitations. The database used is the most dependable data provision platform in China, with highly reliable real-time data; its use also avoids response rate differences (selection intervals). The database uses international codes for standardizing drugs (ATC and DDD), to make Chinese data comparable to those of other countries.

In terms of limitations, we focused mainly on drug procurement data in the national platform, which excludes retail pharmacies and some medical institutions that do not participate in online data collection. Therefore, the antimicrobial use figures given in this report are underestimated. This report does not contain national data on the use of antimicrobials before 2015, and fails to objectively evaluate the impact of previous policies prohibiting the use of antibiotics. In some areas, the quality of the data on reported drug purchases is not high, and there is a lack of information. We did not evaluate the prescriptions orany other prescribing pattern, thus our study lacksdetails on rational or irrational prescribing. Antibiotics are not differentiated between outpatient and inpatient use. The CDSIP data do not contain information about patients, so it is not yet statistically possible to analyze the characteristics of antimicrobial use for different age groups and specific disease groups.

## 5. Conclusions

We identified a slight decrease in the use of most macrolide and lincosamide antibiotics over time in China, with consistent decreases in erythromycin consumption and slight but steady increases in clindamycin and roxitromycinusage. This overall decrease maybe related to China’s strict implementation of health policies. The amount spent by patients on antibiotics increased, and regional variation was also present, which may be due to the variable patient flow to more developed regions.Greater attention should be paid to the irrational prescribing of antibiotics, and more detailed prescribing information should be provided to evaluate antibiotic use. More in-depth studies are required to optimize antibiotic prescribing and reduce antibiotic resistance.

## Figures and Tables

**Table 1:** Antibiotic consumption by ATC classification in China during 2015–2017.

Classification	2015			2016			2017			
	DDDs	Oral	Parenteral	DDDs	Oral	Parenteral		DDDs	Oral	Parenteral
J01F Macrolides		%	%		%	%			%	%
J01FA01 Erythromycin	1.72	63.8	36.2	1.54	64.8	35.2		1.43	68.9	31.1
J01FA02 Spiramycin	0.94	100	0.0	0.83	100	0.0		0.65	100	0.0
J01FA03 Medicamycin	0.58	100	0.0	0.37	100	0.0		0.33	100	0.0
J01FA06 Roxithromycin	7.75	100	0.0	7.93	100	0.0		8.03	100	0.0
J01FA07 Josamycin	0.072	100	0.0	0.091	100	0.0		0.06	100	0.0
J01FA09 Clarithromycin	5.01	100	0.0	5.33	100	0.0		5.15	100	0.0
J01FA10 Azithromycin	5.04	53.1	46.9	5.45	54.1	45.9		4.83	52.9	47.1
J01FA13 Dirithromycin	1.03	100	0.0	1.23	100	0.0		1.09	100	0.0
J01FN1 Roxithromycin and Ambroxol	0.23	100	0.0	0.31	100	0.0		0.32	100	0.0
J01FN2 Acetylkitasamycin	0.095	100	0.0	0.17	100	0.0		0.16	100	0.0
J01FN3 Kitasamycin	0.032	34.7	65.3	0.028	39.7	60.3		0.019	31.5	68.5
**Lincosamides**										
J01FF01 Clindamycin	1.39	43.0	57.0	1.12	42.7	57.3		1.02	42.9	57.1
J01FF02 Lincomycin	1.23	10.0	90.0	1.16	9.5	90.5		1.02	8.4	91.6

DDD, defined daily dose.

## Data Availability

The data presented in this study are available on request from the corresponding author.
